# Nutritional value and environmental aspects of high-protein ultra-processed foods on the German market

**DOI:** 10.1017/S1368980024001836

**Published:** 2024-10-18

**Authors:** Jana Koop, Svenja Fedde, Franziska A Hägele, Christina Beunink, Manfred J Müller, Anja Bosy-Westphal

**Affiliations:** Institute of Human Nutrition and Food Science, Christian-Albrechts-University Kiel, Düsternbrooker Weg 17, Kiel 24105, Germany

**Keywords:** High-protein, Ultra-processed foods, Nutritional value, Environmental aspects

## Abstract

**Objective:**

To compare nutritional value and aspects with environmental impact of high-protein (HP) and ‘normal-protein’ (NP) ultra-processed foods (UPF).

**Design:**

299 HP and 286 NP products were evaluated regarding aspects of nutritional value, energy density, Nutri-Score, number of additives as well as hyper-palatability and price. Environmental impact of HP UPF was addressed by analysing protein sources and the use of environmentally persistent non-nutritive artificial sweeteners.

**Setting:**

Cross-sectional market analysis in German supermarkets and online shops.

**Participants:**

299 HP and 286 NP UPF products.

**Results:**

HP compared to NP UPF had a lower energy density, a lower content of sugar, total and saturated fat, whereas fibre and protein content (62·2 % animal protein) were higher (all *P* < 0·001). HP products therefore had a higher prevalence of Nutri-Score A (67·2 % *v*. 21·7 %) and a lower prevalence of Nutri-Score E (0·3 % *v*. 11·2 %) labelling (both *P* < 0·001). By contrast, salt content and the number of additives (environmentally persistent sweeteners, sugar alcohols, flavourings) were higher in HP compared to NP UPF (*P* < 0·001). When compared to HP products, twice as many NP were identified as hyper-palatable (82·5 % *v*. 40·5 %; *P* < 0·001). The price of HP was on average 132 % higher compared to NP UPF (*P* < 0·001).

**Conclusions:**

While major adverse aspects of UPF regarding nutritional profile and hyper-palatability are less pronounced in HP compared to NP products, higher salt content, increased number of additives and negative environmental effects from frequent use of animal protein and environmentally persistent sweeteners are major drawbacks of HP UPF.

The increasing popularity of high-protein (HP) products in Germany^(1)^ is driven by consumer expectations like improvements in muscle mass^(2)^ and weight loss benefits^(3)^ due to increased satiety^(4)^ and enhanced energy expenditure^(5)^. These aspects also increase willingness to pay a higher price for the products^(6)^. To be labelled as ‘HP’, products must provide ≥ 20 % of their total energy content from protein^(7)^. HP products might however not be as healthy as consumers expect as the majority are convenience products like sweet or savoury snacks, sweetened dairy products and pre-prepared dishes classified as ultra-processed foods (UPF), according to the NOVA system^(8)^. UPF usually have a high energy density and contain high levels of sugar, salt, saturated fats and low amounts of fibre^(9)^. Several studies have linked high consumption of UPF to an increased risk for non-communicable diseases such as type 2 diabetes, CVD and cancer^(10)^. A major risk factor associated with UPF consumption is a positive energy balance^(11,12)^. Compared to low or unprocessed foods, a high consumption of UPF is associated with an increased *ad libitum* energy intake^(11,13)^ which may be due to their higher energy density, hyper-palatability^(14)^, soft texture^(15)^ and/or a low-protein content of UPF^(9,13)^.

Among other factors, the environmental impact of HP UPF depends on the type of protein source used in these products^(16)^. The production of animal protein (e.g. whey protein) *v*. plant protein (e.g. soya protein) is known to increase the carbon footprint^(16)^. Another aspect is the use of non-nutritive sweeteners in HP UPF^(6)^, which are in part environmentally persistent as they are not readily biodegradable^(for review see^ ^(17))^. Acesulfame K (E 950), cyclamate (E 952), saccharin (E 954), sucralose (E 955) and aspartame-acesulfame salt (E 962) are incompletely metabolised by the human body and released nearly unchanged into the environment^(17)^. Multiple studies demonstrated their widespread distribution in the atmosphere as well as aquatic systems such as surface water, tap water, groundwater, seawater and lakes^(17)^.

This study conducts a market analysis that compares UPF marketed as ‘HP’ to conventional UPF of the same category in German supermarkets and online shops, examining their nutritional value (the content of protein, carbohydrates, sugar, total and saturated fat, fibre and salt, energy density, number of additives, Nutri-Score) and price. The environmental impact of HP UPF is addressed by analysing protein sources and non-nutritive sweetener content.

## Methods

### Data collection and product classification

Data were collected between April and July 2023 in selected supermarket chains and online shops in Germany. The aim was to include all available HP products during this period and to find one or two similar ‘normal-protein’ (NP) UPF products as a reference. The investigation in supermarkets focused on the four leading retail groups, with a collective market share of 76·0 % in 2022^(18)^. These include EDEKA and Netto from the EDEKA Group, REWE and PENNY from the REWE Group, LIDL and Kaufland from the Schwarz Group and ALDI-Nord from the ALDI Group with a market share of 25·3 %, 21·2 %, 18·3 % and 11·2 %, respectively, in 2022^(18)^. Additionally, products from the independent grocery store ‘Citti Markt’ (CITTI Märkte GmbH & Co. KG, Kiel) were incorporated into the analysis. Respective websites of the retailers and online shops, except for online stores exclusively addressing athletes, were also examined. The products were sorted into six categories: cereal products, dairy (and dairy alternative) products, sweet and savoury snacks, convenience products and meat alternatives.

### Selection of high-protein ultra-processed foods and reference products

The selection of items for the market analysis was based on specific criteria. All products had to be classified as UPF as defined by the NOVA classification system^(8)^. The only exception was NP pasta (NOVA 1, *n* 5) due to the absence of NOVA 4 category items. The HP products were required to be enriched with protein and to contain ≥ 20 % of their total energies from protein^(7)^. Only products advertised with terms like ‘HP (content)’, ‘more protein’, ‘protein-rich’ and ‘rich in protein’ were classified as HP products. UPF with labels like ‘source of protein’, ‘with protein’ or ‘based on, e.g. soya protein’ were not defined as HP UPF. Protein powder was not included in the analysis. The reference products were conventional UPF without protein enrichment or ‘HP’ labelling. These NP products were chosen to be comparable to the HP products in terms of labelling and food category. The data collection did not follow a matched-pairs design. However, whenever possible, products were selected from the same brand for direct comparison of HP and NP products. No limitations were imposed on protein content in NP products, as exemplified by some dairy products naturally containing ≥ 20 % of energies from protein. The undiscounted price of the products was assessed as cost per 100 g.

### Nutritional value

The nutritional information was obtained from product package labels and information on the corresponding website. These included the content of energy (kcal/100 g), fat, saturated fat, carbohydrates, sugar, protein, salt and fibre in g/100 g. Energy density was expressed as kcal/g. The percentages of macronutrients (% of energies) were calculated using the following factors: protein (4 kcal/g), carbohydrates (4 kcal/g), fat (9 kcal/g) and fibre (2 kcal/g)^(19)^. The number and use of additives (flavour enhancers, sugar alcohols, steviol glycosides, aspartame, emulsifiers and colouring agents)^(20)^, as well as the fortification with vitamins and minerals^(21)^, the use of flavourings^(22)^, yeast extract and added natural fibre were assessed through examination of the ingredient list.

### Nutri-Score

The Nutri-Score, first implemented in France in 2017, is a front-of-pack label designed to simplify understanding of a product’s nutritional value^(23)^. It features a five-colour scale from dark green to dark orange and corresponding letters A (good rating) to E (poorer rating)^(23)^. Products are categorised on the basis of nutrient content (per 100 g or 100 ml), taking into account both nutrients that should be restricted (energy, saturated fat, sugar and salt) and those that should be encouraged (fibre, protein and fruit, vegetables, pulses, nuts and certain oils)^(23)^. The Nutri-Score was documented for all products with a respective label or accessible information on the website. In cases where no label or information was available, the Nutri-Score was calculated using the Excel sheet with the original algorithm provided by Santé publique France^(23)^. The percentage of fruits, vegetables, pulses, nuts and rapeseed, walnuts and olives was derived by examining the ingredient list. For products without the indication of fibre content, a value of 0 g/100 g was assumed to determine the Nutri-Score.

### Defining hyper-palatable products

Fazzino et al. (2019) developed a quantitative definition of ‘hyper-palatable’ foods^(24)^. These foods are known for their high energy density and ingredients that enhance palatability such as fat, sugar and sodium^(24)^. HP and NP products were categorized into three different clusters of hyper-palatable foods that met one or more of the following criteria: (i) >25% of kcal from fat and ≥0·30% sodium by food weight (FSOD, sodium content calculated with salt per gram divided by 2·5 according to Santé publique France^(23)^), and/or (ii) > 20 % of kcal from fat and > 20 % of kcal from simple sugars (FS, 4 kcal per g, both added and naturally occurring sugars) and/or (iii) > 25 % of kcal from carbohydrates and ≥ 0·20 % sodium by food weight (CSOD). The content of simple sugars (g) was subtracted from carbohydrates (g) before calculating the percentage value of carbohydrates. According to Fazzino et al. (2019), the % kcal from carbohydrates used in the hyper-palatability assessment is calculated without simple sugars and fibre^(24)^. In our analysis, we only subtracted simple sugars from carbohydrate content because fibre content was only available in a subgroup of 321 products (HP: *n* 181, NP: *n* 140). In addition, carbohydrate content of food is indirectly measured by subtracting water, ash, protein, fat and insoluble organic fibre from total mass^(19)^. By contrast, fibre content is measured directly using specific analytical methods^(19)^. The discrepancy between methods led to negative values for % kcal carbohydrates in thirty-two products of our analysis. Therefore, we have chosen not to subtract fibre from carbohydrate content. However, subtracting fibre from carbohydrate content led to a slight and non-significant decrease in the prevalence of hyper-palatable HP UPF (60·5 % *v*. 59·5 %).

### Environmental aspects of high-protein ultra-processed foods

The use of potentially environmentally persistent non-nutritive artificial sweeteners was assessed^(20)^. These include acesulfame K (E 950), cyclamate (E 952), saccharin (E 954), sucralose (E 955) and aspartame-acesulfame salt (E 962)^(17)^. Since aspartame is almost completely metabolised into naturally occurring amino acids (phenylalanine, aspartic acid), along with methanol we categorised it as non-environmentally persistent)^(17)^. The information about the type of supplemented proteins in HP products was obtained from the list of ingredients.

### Statistical analysis

Data were collected, processed and graphically displayed using Microsoft^®^ Excel^®^ 2019 (version 1808, Microsoft Corporation, Redmond, USA). The statistical analysis was conducted utilising IBM SPSS Statistics© 27·0 (SPSS Inc.) with the significance level set at *P* < 0·05. Normal distribution was rejected through the Kolmogorov–Smirnov test. Consequently, the data were presented as median and interquartile range. To assess differences between HP and NP products in energy and nutrient content (protein, carbohydrates, sugar, fat, saturated fat, salt, fibre), number of additives and price, the Mann–Whitney *U* test for two independent samples was used. The frequency of the use of non-nutritive artificial sweeteners, sugar alcohols, flavourings, vitamins and minerals, flavour enhancers and yeast extract in HP *v*. NP products was compared using a chi-Square test. Differences between frequencies of Nutri-Score categories and hyper-palatability clusters were analysed by chi-Square test with adjusted standardised residuals and Bonferroni-adjusted post hoc test. If expected frequencies were < 5 or *n* < 20, Fisher’s exact test was used (use of flavour enhancers, yeast extract, aspartame, a combination of palatability clusters 1 and 2 and 1, 2 and 3).

## Results

In total, 585 UPF products were included in the study. The number of items within each category is shown in online supplementary material, Supplemental Figure 1.

### Nutritional value

Table 1 compares nutrient information between HP and NP products in total and within food categories. Information on fibre content was available for a subgroup of 321 products (HP: *n* 181; NP: *n* 140). Compared to NP products, HP products had lower contents of carbohydrates, sugar and total and saturated fat (all *P* < 0·001). By contrast, salt (*P* < 0·05) and fibre content (HP: 31·8 g fibre/1000 kcal *v*. NP: 12·9 g fibre/1000 kcal; *P* < 0·001) were higher in HP compared to NP products. The percentages of energy content for the macronutrients differed between HP and NP UPF and were as follows: carbohydrates HP: 33·2 (26·0–40·7) % *v*. NP: 54·1 (42·6–64·2) %, fat HP: 22·5 (16·2–33·2) % *v*. NP: 35·0 (18·7–47·2) % and protein HP: 32·3 (26·6–49·4) % *v*. NP: 10·5 (6·4–14·0) %; all *P* < 0·001). In contrast to NP products, HP food items had a lower non-beverage energy density (HP: 2·5 (1·1–3·8) kcal/g *v*. NP: 3·2 (1·7–4·7) kcal/g) and beverage energy density (HP: 0·6 (0·5–0·6) kcal/g *v*. NP: 0·7 (0·6–0·8) kcal/g; both *P* < 0·001).

**Table 1:** Comparison of nutritional information of high-protein and normal-protein ultra-processed foods stratified in six categories (median values and interquartile ranges)

			Energy density		Total carbohydrates		Sugar		Total fat		Saturated fat		Protein		Fibre†		Salt	
			kcal/g		g/100 g		g/100 g		g/100 g		g/100 g		g/100 g		g/100 g		g/100 g	
	Group	*n*	Median	IQR	Median	IQR	Median	IQR	Median	IQR	Median	IQR	Median	IQR	Median	IQR	Median	IQR
Total		585																
	HP	299	2·2	0·8–3·8	17·0	6·5–30·0	3·7	2·1–4·9	5·8	1·6–12·0	1·1	0·8–2·8	16·3	10·0–30·0	7·0	5·0–13·8	0·5	0·2–1·3
	NP	286	**2·8** ***	1·3–4·5	**42·2** ***	16·0–57·0	**10·0** ***	3·5–20·1	**7·3** ***	3·2–21·0	**2·6** ***	1·0–6·8	**6·2** ***	3·4–8·5	**3·6** ***	2·4–6·1	**0·4** *	0·1–1·1
Cereal products		142																
	HP	70	2·5	2·1–3·3	21·0	15·0–31·3	2·0	0·6–3·2	6·0	2·8–8·9	1·0	0·5–2·1	19·0	14·1–25·3	10·4	6·1–27·6	1·1	0·3–1·4
	NP	72	**3·2** ***	2·6–3·6	**50·2** ***	42·4–56·0	**3·9** ***	2·7–17·8	5·0	2·4–12·0	1·0	0·5–3·3	**8·5** ***	7·3–10·0	**5·2** ***	3·0–9·5	**1·0** *	0·4–1·2
Dairy/dairy alternative		196																
products	HP	103	0·8	0·6–0·9	6·0	5·0–8·7	4·5	4·0–4·9	1·5	0·9–1·8	0·9	0·6–1·1	9·6	7·5–10·0	1·7	0·7–5·7	0·2	0·1–0·2
	NP	93	**1·0** ***	0·8–1·4	**14·0** ***	10·7–17·0	**11·8** ***	10·0–13·4	**3·7** ***	1·9–8·1	**2·5** ***	1·0–5·6	**3·2** ***	2·8–3·5	1·0	0·5–1·7	**0·1** *	0·1–0·2
Sweet snacks		98																
	HP	50	3·9	3·7–4·3	30·0	27·0–39·0	3·9	2·6–21·0	16·5	13·0–22·3	8·2	6·3–10·3	30·0	26·0–32·3	6·3	5·0–13·8	0·5	0·4–0·6
	NP	48	**5·1** ***	4·7–5·3	**57·9** ***	49·3–62·0	**38·3** ***	29·2–50·3	**26·5** ***	22·3–32·8	**11·8** ***	8·5–15·0	**6·6** ***	5·5–9·0	**4·2** *	2·3–6·4	0·4	0·2–0·6
Salty snacks		58																
	HP	29	4·2	4·0–4·4	34·0	19·0–41·5	2·7	2·1–3·2	12·0	11·0–16·0	1·2	1·0–2·9	39·0	31·0–41·0	11·0	8·4–14·0	2·5	1·9–3·1
	NP	29	**5·0** ***	4·8–5·2	**58·0** ***	53·0–65·2	**3·7** *	2·5–4·9	**24·0** ***	21·2–28·5	**3·0** ***	2·4–6·5	**6·6** ***	6·1–7·8	**3·9** ***	3·1–4·5	**1·7** ***	1·3–2·0
Convenience products		60																
	HP	27	3·6	1·3–4·0	19·0	11·0–45·0	4·0	3·0–8·3	6·4	4·1–8·9	2·2	1·1–2·5	25·0	8·0–41·0	5·0	2·9–7·0	1·3	1·0–2·8
	NP	33	3·2	1·5–3·8	**49·3** **	18·5–63·4	3·2	2·4–5·3	5·7	3·1–9·7	2·0	1·6–3·6	**8·5** ***	6·2–11·8	3·1	1·9–5·1	1·1	0·7–3·5
Meat alternatives		31																
	HP	20	2·0	1·6–2·2	7·6	3·7–12·5	1·5	0·6–2·3	10·0	7·4–11·0	0·9	0·7–1·3	16·0	12·5–21·3	6·2	3·6–7·4	1·3	1·0–1·6
	NP	11	2·0	1·7–2·3	**21·0** ***	19·0–25·0	2·1*	1·7–2·8	9·7	7·5–12·0	0·9	0·8–1·5	**6·8** ***	5·9–7·9	4·5	3·0–7·6	1·2	1·1–1·2

IQR, interquartile range. HP, high-protein. NP, normal-protein. *P < 0·05, **P < 0·01 and ***P < 0·001 as assessed by Mann–Whitney U test, *P*-values refer to differences between HP and NP products and are further indicated in bold. †Information on fibre content was available in a subgroup of 321 products (Total, HP: *n* 18, NP: *n* 140; cereal products, HP: *n* 65, NP: *n* 39; dairy/dairy alternative products, HP: *n* 19, NP: *n* 13; sweet snacks, HP: *n* 35, NP: *n* 31; salty snacks, HP: *n* 29, NP: *n* 23; convenience products, HP: *n* 15, NP: *n* 25; meat alternatives, HP: *n* 18, NP: *n* 9.

In HP compared to NP cereal and dairy (and dairy alternative) products, as well as sweet and salty snacks, a lower energy density was observed (all *P* < 0·001), whereas no difference in energy density was found in HP *v*. NP convenience products and meat alternatives (*P* > 0·05). Total and saturated fat content were lower in HP compared to NP dairy (and dairy alternative) products, as well as in sweet and salty snacks (all *P* < 0·001). Across all food categories, HP UPF exhibited a higher protein content and lower carbohydrate content compared to NP UPF (all *P* < 0·001). The sugar content of HP *v*. NP UPF was consistently lower across all food categories (all *P* < 0·001) except for convenience products (*P* > 0·05), with NP sweet snacks showing a nearly ten times higher sugar content compared to HP sweet snacks (*P* < 0·001). Fibre content was notably higher in HP cereal products (HP: 41·6 g/1000 kcal *v*. NP: 16·3 g/1000 kcal), as well as in sweet snacks compared to NP products of the same category (all *P* < 0·001). The salt content was particularly higher in HP compared to NP salty snacks (+0·8 g/100 g; *P* < 0·001), as well as in HP cereal products, dairy (and dairy alternative) products compared to NP food items within the same category (both *P* < 0·05).

### Nutri-Score

**Fig. 1:**
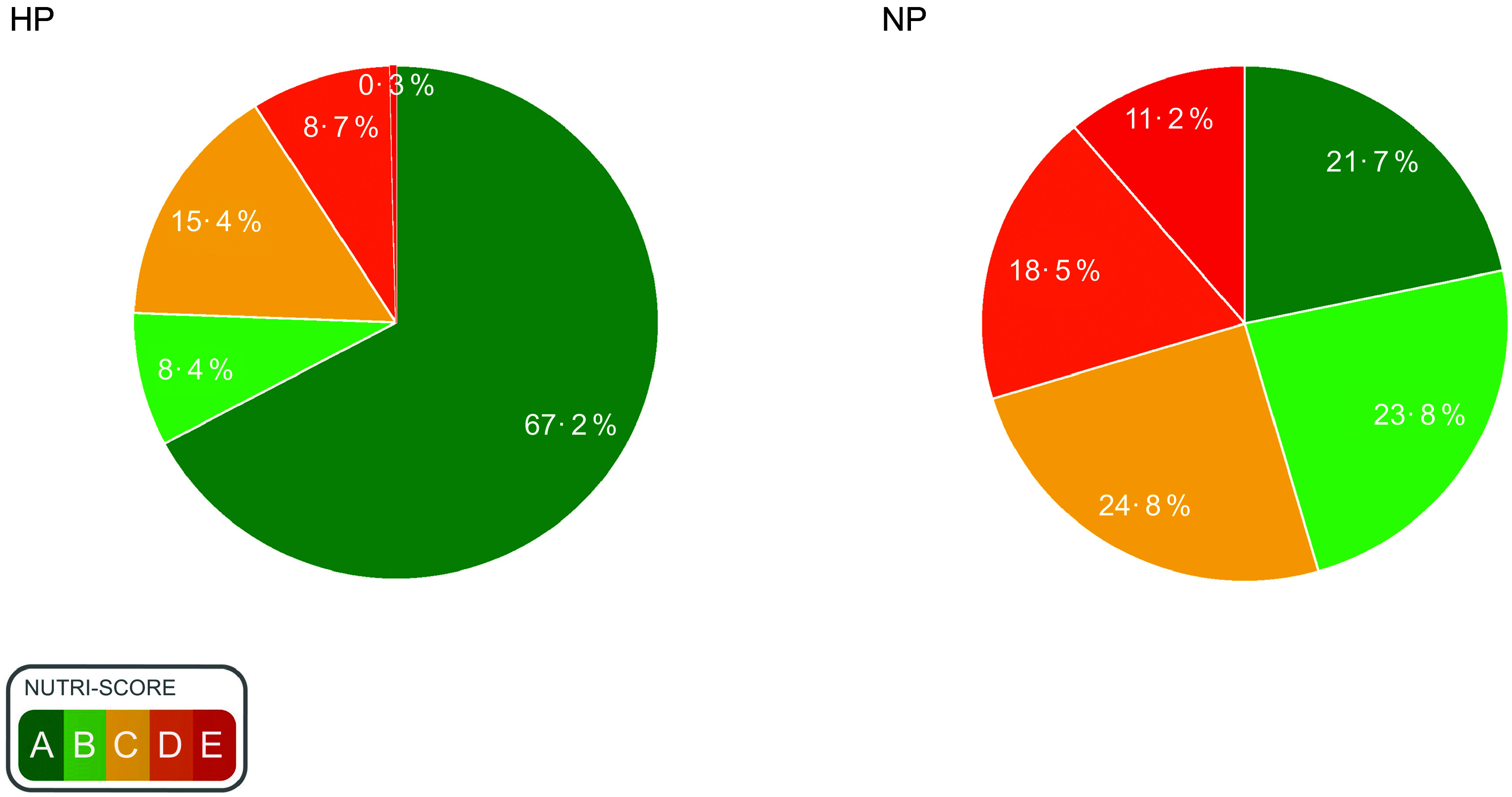
Nutri-Score of high-protein (HP) *v*. normal-protein (NP) ultra-processed foods. Data presented as the percentage of products achieving a specific score. Differences in percentage of HP *v*. NP products with Nutri-Score A, B, C, D and E were all significant (*P* < 0·001)

The prevalence of Nutri-Score A was higher in HP compared to NP UPF (*P* < 0·001, Fig. 1). 67·2 % of HP products achieved Nutri-Score A, followed by C, D, B and E. In contrast, most NP products received a Nutri-Score C, followed by B, A, D and E. Differences in the percentage of HP *v*. NP products with Nutri-Score A, B, C, D and E were all significant (*P* < 0·001).

### Hyper-palatable foods

**Fig. 2:**
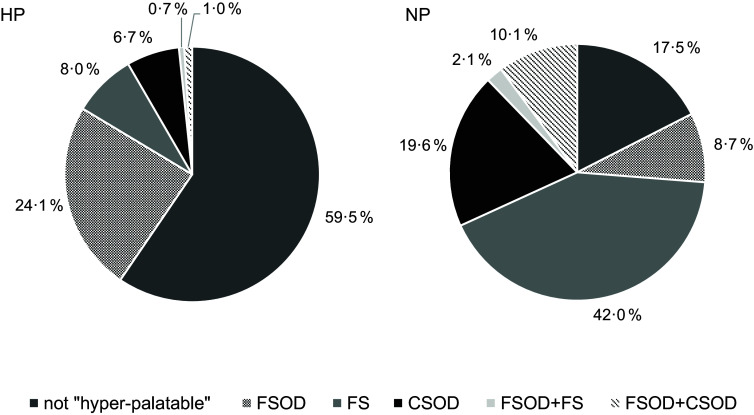
Percentage of hyper-palatable foods (clusters defined by Fazzino et al., 2019)^(24)^ in HP (high-protein) *v*. NP (normal-protein) ultra-processed foods. FSOD (> 25 % kcal from fat and ≥ 0·30 % sodium by food weight), FS (> 20 % kcal from fat and > 20 % of kcal from simple sugars), CSOD (> 25 % kcal from carbohydrates and ≥ 0·20 % sodium by food weight); content of simple sugars was subtracted from carbohydrates before calculating the percentage value of carbohydrates), not hyper-palatable: products (did not meet any criteria of the clusters)

Twice as many NP compared to HP products met at least one criterion for a cluster defining them as hyper-palatable foods (82·5 *v*. 40·5 %; *P* < 0·001). Compared to NP products, HP products were more frequently classified into the cluster FSOD (*P* < 0·001; Fig. 2). By contrast, NP products more frequently belonged to the hyper-palatable clusters FS (*P* < 0·001), CSOD (*P* < 0·001) or the combined clusters FSOD and FS (*P* < 0·001). None of the products met the criteria for the combined clusters FS and CSOD or all three clusters.

Online supplementary material, Supplemental Fig. 2 illustrates the key nutrients that define hyper-palatable foods^(24)^ in both HP and NP products (% of kcal; sodium: % of food weight) showing the higher content of these nutrients in NP products.

### Additives, flavourings and fortification with vitamins and minerals

The number of additives was higher for HP compared to NP products (3 (2–5) *v*. 2 (1–4); *P* < 0·001). In HP products, the use of environmentally persistent non-nutritive artificial sweeteners (*P* < 0·001), stevia (*P* < 0·01), aspartame (*P* < 0·001) and sugar alcohols (*P* < 0·001) was more prevalent than in NP products whereas flavour enhancers were used more frequently in NP compared to HP UPF (Table 2; *P* < 0·05). No differences were observed between HP and NP UPF regarding the use of colourings or emulsifiers (Table 2).

**Table 2:** Utilisation of environmentally persistent non-nutritive artificial sweeteners, aspartame, stevia, sugar alcohols, colourings, emulsifiers and flavour enhancers in high-protein *v*. normal-protein ultra-processed foods

	HP	NP
	*n* 299	*n* 286
	%	%
Environmentally persistent		
Non-nutritive artificial sweeteners†	**34·4**	**0·0** ***
Aspartame	**3·0**	**0·0** ***
Stevia	**6·7**	**0·0** **
Sugar alcohols‡	**25·8**	**0·3** ***
Colourings	12·4	12·2
Emulsifiers	56·5	50·7
Flavour enhancers	**0·7**	**3·1** *

HP, high-protein. NP, normal-protein. *P < 0·05, **P < 0·01 and ***P < 0·001 as assessed by chi-square test, *P*-values refer to differences between frequencies of the utilisation of additives in HP *v*. NP ultra-processed food and are further indicated in bold. ^†^Environmentally persistent according to Lewis and Tzilivakis et al., 2021: acesulfame K (E950), cyclamate (E952), saccharin (E954) and sucralose (E955). ^‡^Sugar alcohols: sorbitol (E420), maltitol (E965), xylitol (E967) and erythritol (E968), none of the products contained: mannitol (E421), isomalt (E953) or lactitol (E966).

73·6 % of HP compared to 57·2 % of NP UPF contained flavourings (*P* < 0·001), whereas no difference was observed between HP and NP products in the prevalence of yeast extract (HP: 7·0 % *v*. NP 7·3 %; *P* > 0·05) or the fortification with vitamins and/or minerals (HP: 10·4 % *v*. NP: 8·0 %; *P* > 0·05). Added plant fibres (e.g. oat/acacia fibre) were more frequent in HP compared to NP products (HP: 20·7 %, NP: 1·0 %; *P* < 0·001).

### Protein source

Online supplementary material, Supplemental Table 1 provides detailed information on protein sources used to fortify HP UPF. Among the HP products, 62·2 % were enriched with animal-derived proteins, primarily milk protein (60·2 %), collagen hydrolysate (6·4 %) and/or chicken egg protein (3·7 %). Over half of the products (54·8 %) were fortified with plant-derived proteins, such as soya (24·7 %), wheat (24·1 %) and/or pea protein (17·7 %). The most common combinations of plant and animal proteins were milk and soya protein (8·4 %) or milk and wheat protein (2·3 %). Excluding dairy products, the analysis showed that the majority (82·0 %) of HP products were fortified with plant protein, while 43·0 % were fortified with animal protein.

### Price

On average, the price of HP UPF was 132 % higher than for NP UPF (HP: 1·9 (0·8–3·6) €/100 g *v*. NP: 0·8 (0·4–1·5) €/100 g; *P* < 0·001). Prices varied across subcategories which are summarised in online supplementary material, Supplemental Table 2. Price differences were seen for all subcategories except for HP *v*. NP meat alternatives (*P* > 0·05).

## Discussion

The study aimed to compare the nutritional value of HP with NP UPF and to examine the environmental aspects of HP products. HP UPF generally exhibited a lower energy density, a lower content of sugar, total and saturated fat as well as a higher fibre content when compared to NP products. Due to these facts and the higher protein content, more HP products were labelled with Nutri-Score A. As a further positive aspect, only 40·5 % of HP products met the criteria for hyper-palatable foods^(24)^, compared to 82·5 % of NP UPF. However, salt content and the number of additives like non-nutritive artificial sweeteners were higher in HP products. In addition, the price of HP was on average 132 % higher compared to NP products. Animal-derived proteins (62·2 %) were the major protein source in HP products.

### Nutritional value and health impacts of high-protein ultra-processed foods

The lower sugar and saturated fat content of HP compared to NP UPF might contribute to a lower UPF-associated risk for non-alcoholic fatty liver disease^(12)^, type 2 diabetes^(25)^ and CVD^(26)^. The higher content of salt in HP compared to NP salty snacks is, however, a negative aspect in terms of a cardiovascular risk perspective^(27)^. The reasons for a higher salt content of HP UPF might be technological (e.g. increased shelf-life even at a lower sugar content or effects on the solubility of proteins)^(28)^. Additionally, salt has flavour-enhancing properties^(27)^ and could thus compensate for the lower fat and sugar content of HP UPF. Innovations for sodium reduction in processed foods either substitute salt with soya sauce in processed meat products or combine fermented flavour enhancers (modified from soya sauce) with potassium chloride^(29)^. HP compared to NP ultra-processed cereal products were higher in fibre (41·6 *v*. 16·3 g/1000 kcal). According to the German Nutrition Society, the recommended fibre intake is ≥ 14·6 g/1000 kcal for women and men^(30)^. HP ultra-processed cereal products could thus significantly contribute to the prevention of obesity, CVD and type 2 diabetes due to the positive effects of fibre^(31)^. Although HP sweet and salty snacks also had a higher fibre content compared to NP UPF (see results), these food categories should only have a minor contribution to total energy intake and are therefore not suitable as a healthy fibre source. Some dietary fibres have been shown to increase satiety potentially by fermentation products that lead to endocrine feedback from the colon or by being viscous, increasing oral processing time as well as slowing gastric emptying^(31)^. However, compared to minimally processed foods UPF generally have a softer texture that contributes to a higher eating rate and energy intake^(15)^ and may thus compensate for the positive effect of fibre on satiety. It is important to note that data for fibre content were only available for a subgroup of 321 products (HP: *n* 181; NP: *n* 140). Due to the satiating effect of protein^(32)^, a lower content of protein in conventional UPF^(9,13)^, which we also found in our market analysis (NP, protein content: 10·5 % of kcal from protein or 6·2 g protein/100 g), could pose a risk for overconsumption of UPF. In addition, protein leverage^(33)^ might also contribute to the overconsumption of low-protein UPF. According to the protein leverage hypothesis by Simpson and Raubenheimer, humans prioritise protein when they are forced to trade off protein intake against that of carbohydrate and fat on nutritionally unbalanced diets and a low-protein content will thus lead to overconsumption of energies to meet the protein target^(33)^. On the other hand, according to this hypothesis, a HP consumption above the protein target should lead to a negative energy balance^(33)^. The HP content of HP UPF in our market analysis (32·3 % kcal from protein or 16·3 g protein/100 g) could thus contribute to the prevention of weight gain. This is supported by a meta-analysis of 38 studies that showed a negative association between dietary protein and total energy intake, irrespective of protein replacement by carbohydrates or fat^(34)^. By contrast, an increased protein intake beyond recommended levels did not enhance weight loss in the general population but prevented a decrease in lean mass during weight loss^(35)^ and enhanced muscle mass in combination with resistance training in young adults^(35)^. A secondary analysis^(14)^ of data from two randomised crossover studies, which investigated predictors of ad libitum energy intake, found that protein content was positively associated with meal energy intake during both ultra-processed and unprocessed diets^(14)^. This unexpected finding may be attributed to additional positive predictors of energy intake such as hyper-palatability and energy density, as identified in the secondary analysis^(14)^.

Except for HP convenience and HP meat alternative products, HP products exhibit a 22 % lower non-beverage energy density compared to NP products, which could be beneficial for the prevention of overconsumption^(36)^. However, the energy density of the non-beverage HP products remains relatively high at 2·5 kcal/g compared to unprocessed foods at 1·1 kcal/g^(8)^. This raises uncertainty regarding whether the reduction of energy density observed in HP compared to NP products at 3·2 kcal/g is adequate to decrease overall energy intake. In addition to a high energy density, hyper-palatability of UPF based on % energies from fat, carbohydrates and sugars as well as % sodium by weight^(24)^ is positively associated with increased energy intake^(14)^. Only half as many HP compared to NP UPF were classified as hyper-palatable (see results), due to their lower content of carbohydrates, sugar and fat. HP products were however more frequently classified into the cluster FSOD when compared to NP products (24·1 % *v*. 8·7 %). This is mainly due to higher salt content, especially in salty snacks (HP salty snacks: 2·5 g/100 g *v*. NP: 1·7 g/100 g). However, the hyper-palatability of HP products might be underestimated because the definition of hyper-palatable foods^(24)^ does not include non-nutritive artificial sweeteners, sugar alcohols and flavourings, which were all found to be more prevalent in HP compared to NP UPF. There is some evidence that these additives might contribute to an increased energy intake^(37,38)^. HP compared to NP UPF exhibit a higher prevalence of additives such as flavourings which may be attributed to these additives compensating for the flavour-enhancing properties of fat, which is lower in HP products. The use of additives in UPF may be due to cost-saving objectives of manufacturers through the substitution of less processed and more expensive ingredients, e.g. using strawberry flavour instead of fresh fruit. There may be adverse effects of some additives like emulsifiers^(39)^ or non-nutritive artificial sweeteners^(38)^ on the gut microbiome (e.g. saccharin or sucralose impairing glycaemic responses) as well as potential ‘cocktail effects’ by the combination of various additives^(40)^, topics which are all discussed controversially. In order to reduce the need for artificial sweeteners in UPF, the ‘Novel Sweets project’ by the Federal Ministry of Food and Agriculture aims to optimise the protein structure of naturally occurring sweet-tasting proteins such as monellin, thaumatin, brazzein, curculin and mabinlin (derived from tropical plants) for use as novel food additives^(41)^.

### Potential risks of a high-protein intake

Although the European Food Safety Authority has not set any upper tolerable intake level for protein^(42)^, there is concern regarding potential risks linked with HP consumption such as the association of a HP diet (21 *v*. 15 % kcal from protein) with an increased risk of type 2 diabetes^(43)^. This might be confounded by the fact that high meat consumption often serves as an indicator of an unhealthy dietary pattern characterised by increased intake of SFA, trimethylamine-N-oxide, salt and nitrite^(44)^, as well as other lifestyle factors such as physical inactivity, alcohol consumption and smoking habits^(45)^. However, there is insufficient evidence that a higher intake of animal protein increases the risk of type 2 diabetes or a higher intake of plant protein may reduce this risk^(43)^. An umbrella review conducted recently found no evidence that a protein intake of up to 3·3 g/kg of body weight or 40 % kcal from protein elevates the risk for kidney diseases^(46)^. Nevertheless, most of the included studies were conducted over relatively short-term periods, so a possible long-term risk cannot be assessed^(46)^. There is also inadequate evidence for higher all-cause mortality as a consequence of HP consumption^(47)^.

A consumer preference for HP UPF products (e.g. 1 serving of HP muesli, pizza, protein bar and chocolate pudding per day) integrated into a regular mixed diet, would result in an average protein intake of approximately 2 g protein per kg body weight, resembling about 26 % energy intake from protein (men: 140 g protein/d, women: 120 g of protein/d). Intervention studies are needed to explore the long-term health effects of HP UPF consumption. Data from the German National Nutrition Survey II showed no evidence of insufficient protein intake among the general population, with median intake levels of 64 g/d for women and 85 g/d for men^(48)^. The German Nutrition Society indicates that even individuals with higher protein requirements, such as athletes or older individuals, can meet their targets through conventional protein-rich foods like dairy products, meats, fish or legumes, indicating that consumption of HP UPF is not necessary to meet protein requirement^(49)^.

### Environmental aspects of high-protein ultra-processed foods

The predominant fortification of HP UPF with animal protein at 62·2 % (60·2 % is milk protein) raises concern about the sustainability of these products due to a higher carbon footprint and land-use changes associated with the production of animal protein^(16)^. In line with these considerations, the EAT-Lancet Commission has proposed the ‘Planetary Health Diet’, recommending plant-based foods as the primary source of protein^(50)^. When dairy products are excluded from the analysis, animal protein is still detected in 43·0 % (milk protein: 42·0 %) of the products. The fact that milk protein compared to other animal protein sources is relatively inexpensive due to subsidies, and the prices of HP compared to NP products being twofold higher (see results), may lead to higher profit margins for the food manufacturers and retailers.

The frequent use of non-nutritive artificial sweeteners in HP UPF is another negative aspect, due to their persistence in water and the atmosphere^(17)^. However, evidence of adverse effects of non-nutritive artificial sweeteners on aquatic species is limited, and some studies suggest that saccharin can be removed by certain wastewater treatment methods^(17)^. There is however a need for continuous monitoring and the development of improved wastewater treatment methods to regulate these emerging contaminants effectively.

### Conclusions

Major adverse aspects of UPF, except for salt, and the number of additives, which have negative health impacts, are improved in HP compared to NP products due to their lower energy density, reduced content of saturated fats and sugars, and higher fibre and protein content. However, protein intake in the population is sufficient and the higher prices of HP products compared to NP products are not justified. Besides these findings, the negative effects of HP UPF on the environment due to the predominant use of animal protein and higher content of environmentally persistent sweeteners are major drawbacks of HP UPF. Intervention studies are necessary to investigate the long-term effects of HP UPF consumption on health outcomes like weight gain.

## Supporting information

Koop et al. supplementary material Koop et al. supplementary material

## Supplementary material

For supplementary material accompanying this paper visit https://doi.org/10.1017/S1368980024001836
