# Supplementary material for: Nutritional value and environmental aspects of high-protein ultra-processed foods on the German market
Source: Public Health Nutr. 2024 Oct 18;27(1):e211. doi: 10.1017/S1368980024001836 (PMC11604323; doi:10.1017/S1368980024001836)
Supplement: Koop et al. supplementary material [file S1368980024001836sup001.pdf]

Nutritional value and environmental aspects of high-protein ultra-processed foods on the German market

Supplementary material

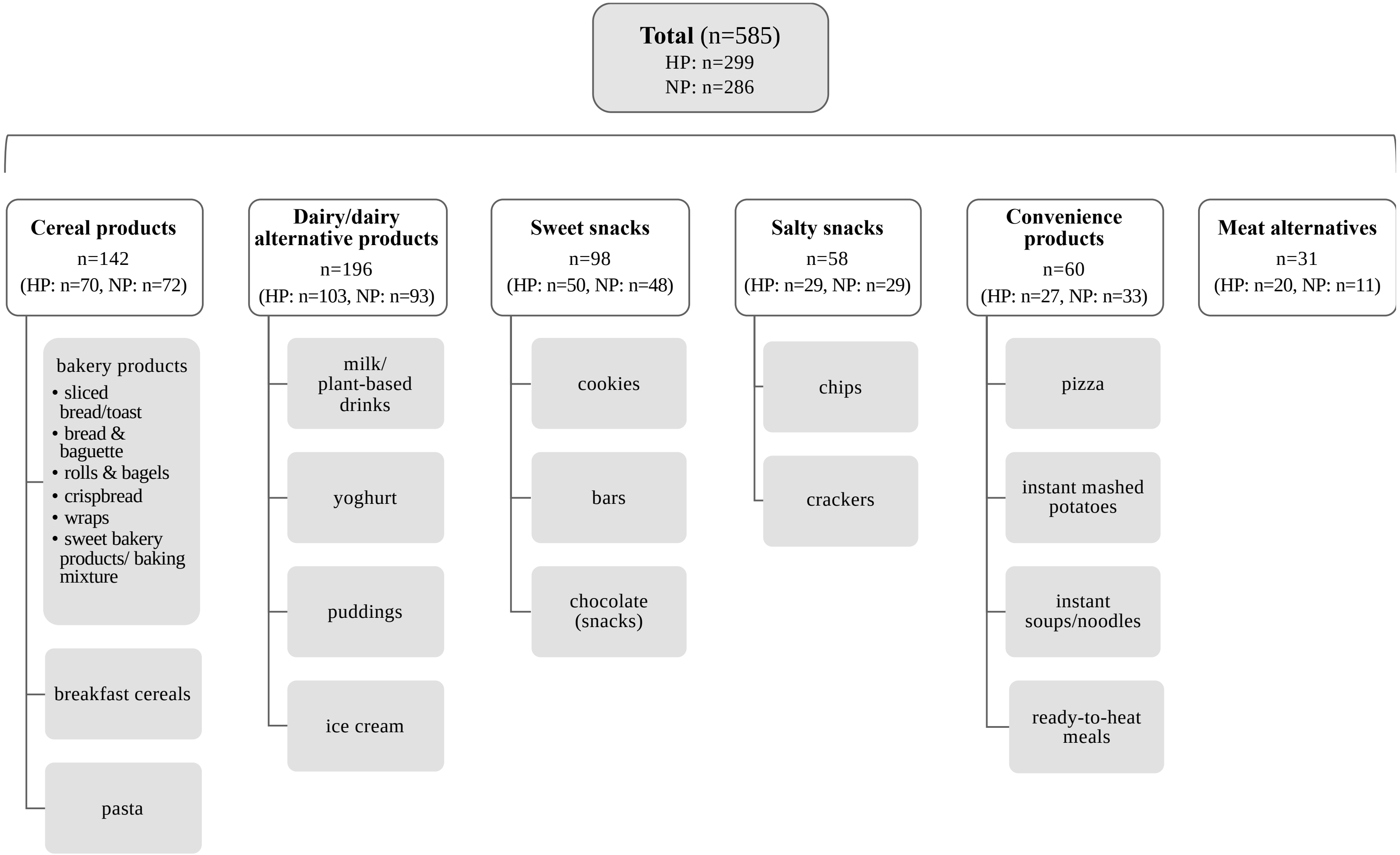

Supplemental Figure 1. Stratification of HP (high-protein) and NP (normal-protein) ultra-processed foods in six food categories

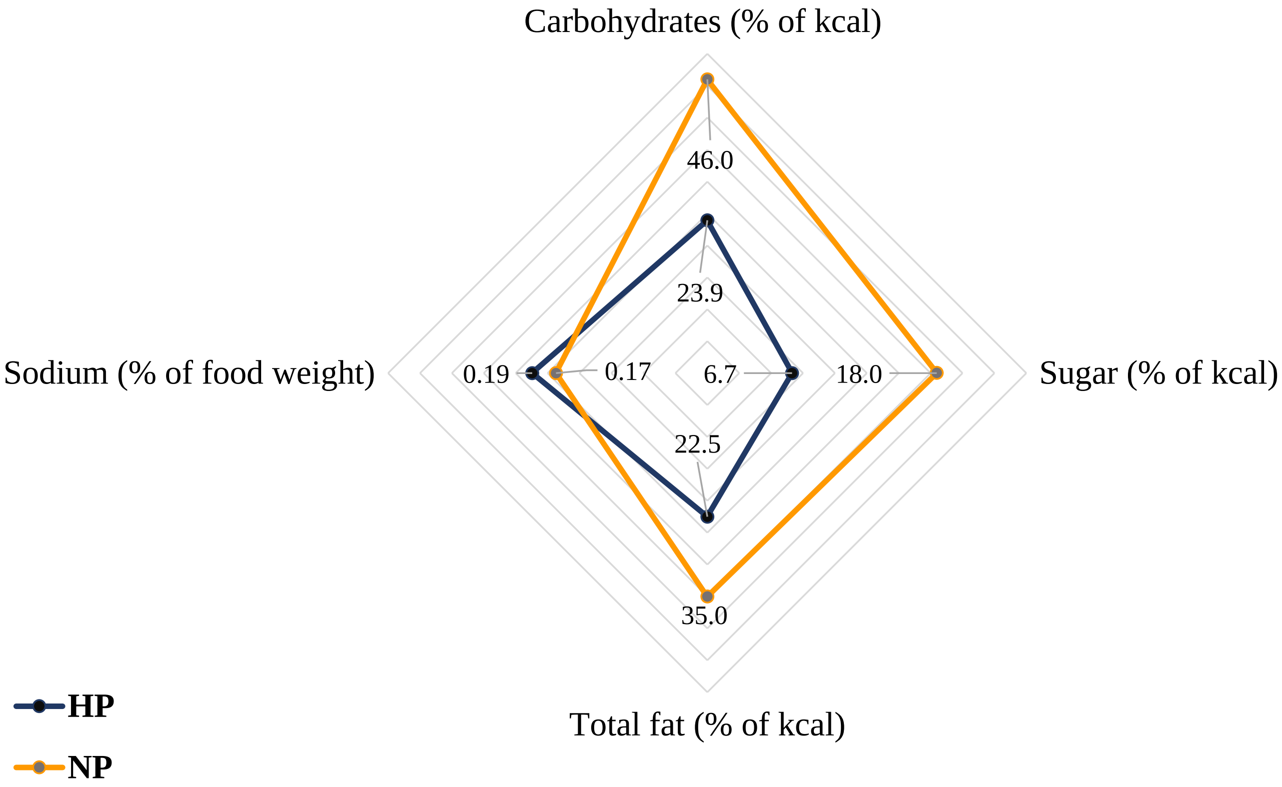

Supplemental Figure 2. Spider Web Plot showing the relevant nutrients for defining hyper-palatable foods according to Fazzino et al. (2019)[24] in high-protein (HP, n=299) vs. normal-protein (NP, n=286) ultra-processed foods, fibre content was not substracted from % of kcal from carbohydrates.
Data presented as median.

Supplemental Table 1. Protein fortification sources in high-protein ultra-processed food.

Protein source	All Products n=299 %	Non-dairy products n=196 %
**Animal-derived†**	**62.2**	**43.3**
milk	60.2	41.8
collagen (hydrolysate)	6.4	8.7
chicken egg	3.7	4.6
**Plant-derived†**	**54.8**	**82.6**
soya	24.7	36.7
wheat	24.1	36.7
pea	17.7	27.0
rice	5.4	8.2
sunflower	3.7	5.6
oat	1.7	2.6
potatoe	1.3	2.0
lupine	1.3	2.0
corn	1.0	1.5
buckwheat	1.0	1.0
rapeseed	0.7	1.0
pumpkin seed	0.7	1.0
**Combination of plant and animal protein**	**17**	**26.0**
milk/soya	8.4	12.8
wheat/pea	4.3	6.6
milk/collagen (hydrolysate)	3.7	4.6
soya/wheat	3.0	4.6
milk/wheat	2.3	3.6
milk/collagen(hydrolysate)/soya	2.0	3.1
pea/rice/sunflower	2.0	3.1
milk/pea/wheat	1.3	2.0
milk/chicken egg/wheat	1.0	1.5
chicken egg/soya/wheat	1.0	1.5
pea/rice/wheat	1.0	1.5
pea/rice/buckwheat	1.0	0.0
pea/rice	0.7	1.0
wheat/lupine	0.7	1.0
milk/chicken egg/collagen(hydrolysate)	0.7	1.0

†combinations of protein sources possible.

Supplemental Table 2. Comparison of price of high-protein and normal-protein ultra-processed food in total and stratified in six food categories.

	Group	*n*	Price €/100 g	
			median	IQR
**Total**		585		
	HP	299	1.9	0.8-3.6
	NP	286	**0.8*** **	0.4-1.5
**Cereal products**		142		
	HP	70	1.7	1.3-2.5
	NP	72	**0.6*** **	0.4-0.9
**Dairy/dairy alternative products**		196		
	HP	103	0.6	0.5-0.9
	NP	93	**0.5*** **	0.3-0.6
**Sweet snacks**		98		
	HP	50	4.1	3.3-4.9
	NP	48	**1.5*** **	1.2-2.1
**Salty snacks**		58		
	HP	29	4.8	3.9-6.0
	NP	29	**1.5*** **	1.3-2.0
**Convenience products**		60		
	HP	27	3.6	2.0-3.7
	NP	33	**1.0*** **	0.6-1.8
**Meat alternatives**		31		
	HP	20	1.7	1.6-2.1
	NP	11	1.8	1.2-2.2

IQR, interquartile range. HP, high-protein. NP, normal-protein. *p<0.05, **p<0.01 and ***p<0.001 as assessed by Mann-Whitney-U-Test, p-values refer to differences between HP and NP products and are further indicated in bold.
